# SELF-REPORTED COMPONENTS OF SELF-IDENTITY IN PERSONS LIVING WITH EARLY-STAGE DEMENTIA

**DOI:** 10.1093/geroni/igad104.0081

**Published:** 2023-12-21

**Authors:** Natalie Regier, Valerie Cotter

**Affiliations:** Johns Hopkins University School of Nursing, Baltimore, Maryland, United States; Johns Hopkins University, Baltimore, Maryland, United States

## Abstract

The impending loss of “self” is a common concern of persons diagnosed with dementia. However, in contrast to the traditional biomedical model, there is qualitative and quantitative evidence that self-identity persists across stages of the disease. An understanding of the components of self-identity of persons living with early-stage dementia (PLWED) can inform treatment approaches and communication from care partners and providers. Consequently, the aim of the present study is to examine what PLWED identify as the facets of their self-concept. Participants were a purposive sample of 20 community-dwelling PLWED who attended a voluntary health program aimed at facilitating aging-in-place. Focus groups were conducted and recorded on-site at the health program. The interviews were transcribed verbatim from the audio recordings and were analyzed through a conventional content analysis approach. Content analysis of the narratives gleaned from focus group participants revealed six self-identity themes: vocation, family role, personal characteristics, hobbies and interests, religious affiliation, and social networks. Although the deterioration of explicit memory, including autobiographical memory, is a hallmark of dementia, overfocusing on this as the sole component of self-identity contributes to the representation of persons living with dementia as less than whole and lacking “selves.” Our findings suggest critical components of self-identity other than memory that should be recognized and supported in persons living with dementia.

